# Challenges encountered by parents from urban, lower social economic class in changing lifestyle behaviors of their children who are overweight or obese

**DOI:** 10.1186/s12887-023-04295-5

**Published:** 2023-09-12

**Authors:** Xiao Ma, Weidong Li, Paul B Rukavina

**Affiliations:** 1 Institute of Sport Sciences, Shanghai University of Sport, Changhai Road 399, Shanghai, 200438 P.R. China; 2 College of Education & Human Ecology, Department of Human Sciences, The Ohio State University, PAES Building A270 305 Annie and John Glenn Avenue, Columbus, OH 43210-1224 USA; 3 Adelphi University, Woodruff hall, 1 South Avenue, Garden City, NY USA

**Keywords:** Obesity, Parental perceptions, Lifestyle behaviors, Urban

## Abstract

**Background:**

Parents from urban, lower social economic classes often encounter unique challenges in their lives, which shape how they work with their children who are overweight or obese to change their exercise and eating behaviors at home. The present study took an initial step to address a gap in the literature by describing the challenges that parents from lower social economic classes in an urban city encountered in changing exercise and eating behaviors of their children who are overweight or obese.

**Methods:**

A conversational style semi-structured interview with prompts and probes was conducted to 44 parents whose child is overweight or obese. Inductive content analysis and constant comparison was used to analyze the data. Data trustworthiness was established by using a variety of strategies.

**Results:**

Two major themes with eight sub-themes emerged from the data: Challenges to promote a healthy active lifestyle, and challenges from their child’s development and lifestyle behavior. Eight sub-themes were: (1) Need for effective strategies for a lifestyle behavior change, (2) monitor and promote healthy choices, (3) money, time, and dangerous neighborhood, (4) scientific knowledge to promote a healthy active lifestyle, (5) developmental changes of adolescence, (6) unmotivated and lack of persistence, (7) sneaking eating, and (8) peer pressure.

**Conclusion:**

The challenges, from economic to parenting, are certainly of importance, and understanding these challenges will be crucial to help school-based professionals develop interventions. Those identified challenges should be clearly placed within family-school collaboration practices.

The Obesity Society 2018 position statement defines obesity as a multi-causal chronic disease [1]. Childhood obesity is associated with a high risk of developing chronic diseases, including cardiovascular disease, cancer, diabetes, hypertension, and musculoskeletal disorders [1], and a risk factor for coronavirus disease [2]. It is also one of the least socially acceptable and stigmatizing experiences for children to have since they often experience psycho-social and emotional damage because of being targeted for teasing and bullying [3, 4]. Childhood obesity progresses into adulthood, and children from urban, lower-income families are at greater risks of developing obesity [5]. The etiology of obesity is multifaceted and complex. In addition to genetics, drug side effects, environmental, and social and cultural factors, unhealthy lifestyle behavior (i.e., physical inactivity and unhealthy eating) is the fundamental cause of overweight and obesity [6]. Parents play a key role of creating a home environment that foster healthy, physically active lifestyle behaviors. They shape their children’s eating and exercise behaviors in numerous ways at different stages of development and growth [7]. Therefore, fostering a healthy active lifestyle through exercise and healthy eating during early and middle childhood through family-based interventions is critical to successfully prevent and reduce the obesity disease.

There is ample research evidence to support the critical role that parents play in their children’s lifestyle behaviors. The findings from correlational studies have shown that parental support, guidance, and behaviors are positively associated with their children’s attitudes [8], eating habits/food consumptions behaviors [9–13], and exercise behaviors [14, 15]. Furthermore, the literature on family- or home-based intervention studies has also shown positive changes in weight reduction and improvement in eating and exercise behaviors [16–18]. The intervention approaches include behavioral modifications, behavioral therapy, and problem solving, targeting nutrition and exercise education, parenting skills, goal setting, management skills, role modeling, self-monitoring, praising, stimulus control strategies, cognitive restructuring, family support, and weight loss maintenance strategies, and problem-solving strategies [16]. Those findings suggest that parents can shape their children’s behaviors through behavioral modeling and parent-child interactions, where they create opportunities of exposure to certain behaviors or actions, share behavioral strategies, experiences, and emotions in dealing with these situations, and provide requisite support for behavior engagement for their child [19, 20].

The family- or home-based intervention on childhood obesity has generally resulted in positive outcomes. However, as Sung-Chan et al. [18]. pindicates, how family dynamics and structure mediate the treatment effects has remained unexplored. Most family-based interventions failed to report participants’ social economic status and have been conducted with middle-class Caucasian youth [16]. Davison et al. [21] also pointed out a limitation in parenting and childhood obesity research. That is, parents from vulnerable populations were under-represented. Recently, Kim and Lee [22] conducted a systematic review on family-based child weight management intervention in early childhood from low-income families. The findings showed an insufficient and inconsistent pattern of results with only four studies demonstrating the effectiveness of reducing body weight. Parents from urban, lower social economic classes often encounter unique challenges in their lives. It is unknown about what challenges that parents from urban, lower social economic classes may encounter in helping their children who are overweight or obese to change their exercise and eating behaviors at home.

The purpose of this study, therefore, was to explore those challenges encountered by parents of children who are overweight or obese from urban, lower social economic classes. A complete understanding of these challenges will be crucial to design effective intervention components and develop effective family-school/community collaboration practices to help parents from urban, lower social economic class to change their child’s unhealthy lifestyle behaviors.

## Methods

The present study used a descriptive qualitative research design. We were interested in the perceptions of barriers and challenges that parents with adolescents who were overweight or obese encountered in helping their children to change their exercise and eating behaviors at home. Those adolescents were from an urban city school district in the Southwestern USA. At the time of data collection, approximately 87% of students in this school district were African American and 71% of students received free or reduced-price lunch.

### Participants

This study was part of a larger one, which investigated weight-related teasing and coping among adolescents who are overweight and obese [3, 23] and parental perceptions and involvement [24]. This study was funded by the Research Consortium—established researcher grant, American Alliance of Health, Physical Education, Recreation and Dance (Currently called Society of Health and Physical Educators). Participants in the present study were 44 parents (42 mothers, 2 fathers) from 42 families (28 African Americans and 16 European Americans) who provided written consent to participate. All participants had children who were overweight or obese with a Body Mass Index (BMI) greater than the 85th percentile adjusted for age and gender calculated from height and weight measures [25]. These adolescents’ BMI ranged from 25 to 62.2 and aged from 11 to 19 years old (M = 14.86, SD = 1.97).

Previous literature had identified overweight and obesity as a sensitive topic [26]. Therefore, we took great care of how to communicate with participants and their families during recruitment. To ensure that we can recruit enough participants with a heterogeneous group, three methods were used. First, we worked with physical education teachers in the city schools to identify a pool of adolescents and their parents. Physical education teachers helped us hand out the assent and parental consent forms to adolescents, who brought them back home to discuss with their parents. If adolescents and their parents were interested in participating in this study, they returned the signed forms. Secondly, we distributed flyers to weight loss clinics identified through internets and summer camps organized by a local community center. The flyers had general information about the study with the contact information of researchers. At the clinics, the supervising physician gave the flyers to the visiting families. If the family showed an interest in the study or had some questions about the study, one of the researchers addressed their questions and provided detailed explanations of this study. If the family decided to participate in the study, they signed the assent and parental consent forms. If the family needed time to think about whether to participate in this study, we provided a phone number for them to contact us. Finally, we recruited participants through “word of mouth” by asking current participants to tell their friends about the study. When interested parties contacted the researchers, more detailed explanations were provided. Consent was obtained if they decided to participate in the study. All the families who participated in the study were awarded a grocery gift card in the amount of $50. An approval from the Institutional Review Board (IRB) for the current study was obtained. Approvals from the school district and school principal, the weight loss clinics, and summer camps were also obtained.

### Data collection

As an appropriate method to explore perceptions of parents with adolescents who are overweight or obese, interviews allowed the researchers to gain insight into the sensitive topic related to obesity with care and have flexibility to address other issues emerged from conversations with parents [27] (Edmunds, 2005). A conversational style interview with follow-up prompts was conducted. The initial questions included: (a) Given that some may see your child as a person who is overweight or obese, as a parent, what do you expect your child to do with their body weight? (b) What are your expectations regarding the type of foods and how much exercise they do? (c) Have you been monitoring your child’s dieting, eating, and physical activity patterns? If so, please elaborate on that.

Each parent participated in one in-depth interview as part of a larger study, which lasted between 30 and 90 min. A face-to-face interview was conducted for 42 parents while two parents participated in a phone interview due to their scheduling constraints. All interviews were conducted by the second and third authors in a private space at participants’ choices to make themselves feel conformable and at ease to talk about those sensitive issues. Both researchers are a university professor in the United States who are well-trained in qualitative research methodology and have experience in conducting interviews and qualitative data analysis. At the start of the interview, researchers engaged in friendly conversations to put parents at ease [28], discussed the significance of this study, and highlighted the importance of making their voices heard. Parents were also assured that they could withdraw from the interview and this study without any repercussions at any time when they felt uncomfortable with the interview. Upon the completion of all individual interviews and transcribing the interviews, a focus group interview was conducted. We invited all the parents to participate in a focus group interview through mail and phone calls. Only six parents volunteered to participate in the focus group interview. During the focus group interview, parents had an opportunity to member-check data interpretations and their views on certain topics were further explored. All interviews were audio-taped with permission from parents.

### Data analysis

Each participant was assigned an ID number and a pseudonym. All audio tapes were transcribed verbatim. Inductive content analysis and constant comparisons were used to analyze the data [29]. The data analysis consisted of three phases. In phase one, two researchers gained familiarity with the data by carefully reading the interview transcripts multiple times. In the second phase, both researchers first determined the unit of analysis, and identified and formed major concepts, which arose frequently from the data, into themes. Then, both researchers developed a coding template with main themes that emerged from the data. To ensure that the operational definitions of themes were clear and consistent, eight interviews were randomly selected and double-coded. The disagreements were discussed between two coders until a 100% consensus was reached. Each coder then independently analyzed the data using the coding template. In the final phase, the researchers made interpretations of the themes and identified the relationships among those themes.

### Data trustworthiness

Five strategies were used to establish data trustworthiness for the present study [30]. First, to ensure a representation of voices from parents with adolescents who are overweight or obese, we recruited 44 parents for our interview. Second, throughout the study and data analysis, we conducted peer debriefing with a colleague who has great expertise and extensive experience in qualitative research. The peer debriefer provided feedback and insights regarding many aspects of this study, including data collection procedures, development of coding template, data coding and interpretation, formation of conceptual linkages, alternate interpretations, overall representation of the data, and how to conduct a focus group interview. Third, transcripts were mailed to all participants for member checks using the address provided at the time of interview. Parents were asked to check the accuracy of transcripts and make any edits as needed. A stamped addressed envelope was provided for parents to mail them back to the researchers. Ten out of 44 parents returned their edits of transcripts. The rest of the mail was returned to us due to incomplete address information. For those returned transcripts, parents only indicated some typographical and grammar errors. Fourth, a focus group interview was conducted to member check the data interpretations. During the focus group interview, the identified themes were presented to participants who checked the accuracy of data interpretations. No new themes were identified from the focus group interview data. Finally, throughout the data analysis, researchers searched for any negative cases to refute themes or provide an alternative point of view.

## Findings and discusson

When working with their child who are overweight or obese on changing his or her lifestyle, however, majority of parents experienced numerous significant barriers and challenges. Two themes with eight sub-themes emerged from the data: Challenges to promote a healthy active lifestyle, and challenges from their child’s development and lifestyle behavior. The eight sub-themes were: (1) Need for effective strategies for a lifestyle behavior change, (2) monitor and promote healthy choices, (3) money and time, (4) scientific knowledge to promote a healthy active lifestyle, (5) developmental changes of adolescence, (6) unmotivated and lack of persistence, (7) sneaking eating, and (8) peer pressure.

### Challenges to promote a healthy active lifestyle

Given the constraints of their adolescent son or daughter who are overweight or obese, parents had a variety of challenges based upon their evaluation of the success they had: Need for effective strategies for a lifestyle behavior change, monitoring and promoting healthy choices, money, time, and dangerous neighborhood environment, and scientific knowledge to promote a healthy active lifestyle.

#### Need for effective strategies for lifestyle behavioral changes

To change their child’s unhealthy lifestyle behaviors, parents had a need for effective strategies. They used a variety of strategies to help them develop and maintain a healthy, physically active lifestyle. They tried to instill a concept of “a healthy body,” educate their child that physical image is changeable, teach responsibility and empower their child with choices, change family diet and monitor it through portion sizes, register their child for sports teams or weight management programs, and exercise with their child. For example, one parent reported that by empowering choices and changing family diet, she was able to successfully help her daughter change unhealthy eating habits and improve self-esteem,

Well, I know that I can’t always be with her. I can’t always be there to have her make good choices so what I did as a parent I changed the way I cooked. I changed the things that we ate, a lot of the things we ate but I also told her it’s up to her to make the right choices when you are at school and at lunch…. it’s also portion control …as a result she’s lost about 65 pounds and I notice it’s made a difference, not only in her appearance but also her self-esteem has shot up.

This parental approach is consistent with Yee, Lwin, and Lau’s concept of active parenting strategies [13]; an active parenting approach influenced three proximal predictors of intention (child’s attitudes, perceived behavior control, and perceived norms) to eat fruits and vegetables. In other words, positively discussing, instructing, and verbally interacting with their child can influence their intention to eat healthy foods.

Another parent tried a variety of strategies to change their son’s unhealthy behaviors. They did “family group walks,” rode bikes together, had a personal trainer work with their child, and registered their child for many sport teams and camps (i.e., basketball, soccer, tennis). However, for many different reasons, such as heat-temperature/weather and loss of interest, they had little success for their son to adhere to these exercise programs. The only successful strategy that the parents perceived worked, was to let their son go on walks on his own with an Ipod. But this activity was not intense enough for his son to lose weight. This parent commented,

We’ve tried um different strategies over the years like I said we’ve gone on family group walks we’ve um rode bikes we have tried to establish an exercise routine it’s been very difficult, the most successful has been he will just go on walks on his own with an Ipod, …… He will take several walks a day. I think the problem is that he is not getting the aerobic workout that he needs because it’s at a slower pace. But I’m very happy with the fact that he is active.

Recently, their son began to work out with a friend under a coach after school. They started to see some positive effects on their son’s energy level. Now school had ended, and his son could not work out with this friend at school. The parents had to register their son for a four-week summer camp so that he can get enough physical activity and they can keep him away from TV, computer, and video games etc. The parents hoped that their son can learn healthy eating and exercise by working with kids with same weight issues and concerns during summer camp.

One of the main reasons he is doing this is because we know if he is at home, he is not going to get the type of physical activity that he needs. But if he is away from home, that means he is away from the TV, computer, and video games…… the camp that we’ve picked out also has a component that teaches the kids about healthy weight loss so I’m very hopeful that this program this summer where he is exposed to a lot of other kids who are struggling with the same weight issue that he is that you know they can come together and learn more of the healthy eating, I think it’s more acceptable to eat vegetables if all the other kids are eating vegetables you know your with and opposed to ‘it’s just your parents eating the vegetables (Laughter).’

#### Monitoring and promoting healthy choices

Some parents were frustrated that schools provided unhealthy meals and their child made bad choices during lunch at school. This was problematic because parents could not monitor their child’s diet like they could at home. Monitoring refers to parents “keeping track” of the consumption of the amounts and types of food [31]. For example, one parent commented,

He definitely eats junk food, and we have to monitor him all the time about that. So, which is a shame, but you know. …When he goes to school, we give him lunch money and he blows that on slushies or hot pockets or whatever you know so and uh there’s just nothing I can do about that. But when he is at home, we always make sure that he is going to eat fruit…. As a family we have a pretty good diet we eat a lot of fresh food and more food from scratch than a lot of Americans ……We bribed him heavily to eat a lettuce leaf once or something. He doesn’t normally ever voluntarily do that.

Another parent said,

Like I said, it’s like when they got into school, it’s like they developed their own pattern of eating, a way of doing things. And um, like I told you, she doesn’t have to metabolism where she can just eat what she wants, and it shows on her body.

Studies on the effectiveness of the relationship of monitoring and children’s diet, eating habits, and weight are inconsistent; some studies show a positive effect whereas others do not [32, 33]. Parenting strategies involving monitoring children’s diets and physical activity levels may be counterproductive [33]. It is hypothesized that there is a curvilinear relationship among the variables; monitoring works up to a single point, but after that it can be counterproductive. Its effectiveness also depends on the characteristics of the individual and circumstances.

#### Time, Money, and dangerous Neighborhood Environment

The high cost of healthy food and busy daily lives are significant barriers that many urban parents with lower social economic status cannot overcome. Almost all the families in the present study were from urban areas and lower income. Some parents worked two jobs to support their family; they did not have time to work with their child on exercises and their incomes were barely able to support the family. Some parents indicated that they did not have time to work with their child on exercise (e.g., they may get one weekend off in three weeks). Even if these parents had time off, they have many other family responsibilities to fulfill, such as repairing houses or vehicles. For example, one parent commented that all she had time to do was to encourage her child to exercise and eat better,

I don’t have a whole lot of time myself because I’m trying to make the repairs on this house, our own vehicles or both. This is my first full weekend off in the last three weeks, so you know between the time when I come in and she is here. I try to get her to do what she should be doing and maybe a little more than what she should be doing. Sometimes, you know to encourage her to work on her own behalf, but you know I’m like so many other parents now days in terms of my work life. … but the time I’m able to spend with her or around her is either exercising her thumbs (playing video games on the phone) or her elbow bringing that fork to the mouth.

Another parent indicated that both she and her husband were working, and it is very hard to put together a healthy meal after coming back home from work. Very often their child stayed at home alone and they ate cookies and chips stored in the cabinet. This parent said,

You know it’s hard, it’s hard to work all day and you know and come home and take care of a family and put together a healthy meal…. There are cookies in the cabinet and chips and things like that. With people’s lifestyle now with the moms and dads working, a lot of kids must stay home by themselves after school. They go in the cabinet and eat what they want to.

Besides busy working lifestyle, some parents indicated that they could not afford to buy healthy food as it is costly and expensive. Making healthy eating choices is important for one’s health and wellbeing. However, for many low-income families, they could not make enough money to buy healthy food for their child. For example, parents commented,

I know she should eat healthy food. I don’t know what type of healthy food, you know she pretty much eats what my wife cooks and you know I mean we’re not able to afford what the law says that’s best for you, you know we just can’t afford.

Another parent had a set budget for grocery shopping and could only buy what they could afford. They wished that the prices for healthy food could come down so that they could purchase for their child. She commented,

You go into the store and try to buy healthy food. It’s more expensive. …… We try to change over to fruits, vegetables, raisins, yogurt, salads, and wheat bread. It’s more expensive. A lot of parents like me, I mean I have three other children and I have so much money to go to store. That’s what I have. I think if the prices came down that would help. I think prices and busy lifestyle all affect the child.

Another issue for lower income families is the lack of available income for health appointments, which could help their child. One family could not even afford taking their child to see a doctor and check out why she was obese. This parent commented,

We don’t have a lot of money. I can’t take her to the doctor and have her checked out to see if it’s genetic or if it’s just her sedentary, or at least partially sedentary lifestyle, you know, playing video games and watching movies.

Social features of environments, such as neighborhood safety, affect children’s and adolescents’ physical activity levels [34]. The neighborhoods where participants in the present study lived were dangerous for their child to even walk on streets. One parent indicated that “I don’t feel comfortable letting her run around the neighborhood freely because of the bad environment.” Another parent called the surrounding neighborhood as a “nut bush.” There were gun fires all the time on the streets and people stole things by smashing car windows. As a result, the researcher had to stop the interview and pulled his car into the driveway rather than parked on the side of the street. This parent commented,

That’s fine, I don’t blame you for being concerned about your car. The other side of Jackson is what we call nut bush, and uh we call it that because of all the nuts that live over there. (There were) gun fire all the time. People stole things by smashing the car windows. Unfortunately, they are moving over to this side of Jackson. That plus the illegals moving in on us.

For these families who do not have time for their child, cannot afford to buy healthy food, and live in a dangerous neighborhood, parents need to identify strategies that can work for them to develop and maintain a healthy, physically active lifestyle.

#### Scientific knowledge to develop a healthy active lifestyle

Even though parents of adolescents who are overweight or obese in the present study recognized the importance of healthy dieting and exercise, expected their adolescents to eat healthy and exercise, and tried to foster a healthy lifestyle through eating healthy and exercise, some of them lacked knowledge on the amount of exercise needed for a healthy weight, how to create a detailed systematic plan, and the persistence to monitor and regulate their behaviors. One parent commented,

She walks twenty-five minutes a day she walks twenty-five minutes to where she’s going and twenty-five minutes back home from where she’s coming from, then she plays basketball sometimes with her brothers or other kids and stuff like that so she’s getting plenty of exercise. Right now, I think she’s getting enough, I think twenty-five minutes is quite a bit enough for walking, so I think that’s a lot.

Parents exercising with their child who is overweight together is an excellent way to model a healthy, physically active lifestyle. However, parents themselves had difficulty in adhering to an exercise program that they designed with their child for many different reasons: being tired, too much homework for their child, and unmotivated. One parent reported “I try but I’m not good at it. We had like, promised each other, a new year’s resolution, of being on treadmill for three hours a week, but we’re not doing it.” Another parent tried to walk with her child. However, “He won’t go every night, but I try to get him to go with me when he will.” They walked together for maybe twenty, thirty minutes four times a week. The parent likes her child to “walk two miles every night with her. But that is not going to happen……he has too much homework, is too tired, and just does not want to do it.” The failure can further reinforce unhealthy behaviors of adolescents who are overweight, thus making future lifestyle interventions more difficult. It is important to provide parents with effective motivational strategies to improve their adherence to an exercise program.

### Challenges from their child’s development and lifestyle behavior

Parents were presented with many different challenges to promote a healthy active lifestyle that are specific to the individual development and lifestyle behavior of their child in attempting to promote a healthy lifestyle. They were the developmental changes of adolescence, unmotivated and lack of persistence, sneaky eating, and peer pressure.

#### Developmental changes of adolescence

Adolescence is a period of rapid growth and change in the areas of physical, intellectual, psycho-social, and emotional development. Adolescents become increasingly independent, and search for adult identity and peer acceptance. These intellectual, social, and emotional changes presented some challenges for parents to work with their child who is overweight to change their unhealthy behaviors. Some parents indicated that adolescents are teenagers who do not listen to their parents anymore. For example, some parents commented,

She does not follow some basic dietary rules and um we’ve tried to explain to her certain things, you need to do this, you need to do this, um but no she doesn’t want to hear it from us.

Well, I’m hoping that he will get some basic nutritional food. We talk our ears off about it, but he doesn’t listen to us because you know sometimes when you have another person come in and tell you something like another adult …… I think teenagers just tune their parents out.

#### Unmotivated and lack of persistence

Another challenge that parents had was that their children lacked motivation and persistence from their child. Being motivated and persistent is a key factor for successful lifestyle behavior change. Parents reported that their child was unmotivated to change their unhealthy lifestyle or tended to “slack off” and had difficulty sticking with an exercise program even after they had experienced some success in losing weight. Some adolescents who are overweight were unmotivated or not persistent because they felt helpless in changing their eating and exercise behaviors or their interests had changed, or they lost their support system from their friends. For example, one parent commented,

I said, ‘the more you try to watch your diet and exercise the more you would burn the weight off.’ But it’s this thing where he gets to the point where he gets discouraged. You know he doesn’t feel like he would stay up with it so he doesn’t do it.

Another parent also commented,

This is a pattern that I see with her. She says she gonna be on a serious exercise program. Then when she loses a little bit of weight, she tends to slack off a little bit…. It seems like I’m always paying a tuition fee and investing money on all these certain kinds of uniform and fitness equipment for some sports for her. If one of her friends or whoever she’s going with doesn’t like it then she quits too.

Apparently, parents struggled with motivating their children who are overweight to engage in a healthy lifestyle and maintaining persistent and committed. They need effective strategies to deal with their children’s lifestyle behavioral changes.

#### Sneaking eating

Parents reported the eating behavior of their children who are overweight presented them a challenge. One specific behavior was that their children were “eating behind their backs”, an eating behavior that they titled “sneaky eating”. Sneaky eating is one of the criteria for diagnosis binge eating disorder in the Diagnostic and Statistical Manual of Mental Disorder (DSM-5), and it is seen in 33% of obese children who are diagnosed with binge eating [35]. Other binge eating symptoms are an inhibition or embarrassment in eating in front of others or eating in absence of hunger. In the present study, those children that exhibited binge eating made it very difficult for parents to work with their child to change their eating behaviors. For example, one parent commented,

She does a lot more secretive eating, not just around me, you know I see her eating a little junk here and there. She kind of eat secretly you know like what we made at the table she don’t eat much in front of me, but I don’t know if she eating more later like at night or something when nobody sees her eating you know because usually I have something she’ll turn it down I know it’s something she wants, she’ll say no.

This loss of control as well as behaviors of using food for comfort, pleasure, or entertainment is indicative of what Laurent argues as these children have a dysfunctional and intimate relationship with food that is enmeshed in their lives [36]. Another parent talked about a conversation she had with her child’s godmother who said, “maybe she has an eating disorder, she loves food, you can just see it in her eyes”. Laurent argues that trying to instill a mentality of “eating less and exercising more” will not be effective in these children, and that parents should investigate the relationship that the obese child has with food [36].

#### Peer pressure

Adolescents who are overweight can experience a lot of peer pressure. Peer pressure is synonymous with the concept of ‘conformity’ where adolescents have pressure to go along with the behaviors and opinions of their friends [37]. These peer pressures can have a negative impact on adolescent’s eating and exercise behaviors [38]. As one parent commented,

I think it’s very difficult for them because they want to engage in all the guilty pleasure that all their friends do. They have a lot of peer pressure, and they want to go to all the social activities they friends do, which usually involves going to the pizza places …… all these places where they have fat foods. That’s just mostly their whole social circle.

Another parent commented that peer influence has a lot to do with his or her children’s struggle to maintain a healthy weight. The parent suspected that her child’s trouble with weight has a lot to do with the influence of friends in a different context than their family house.

She’s big one minute then she’ll come back small and she’ll lose weight here and it’s like an up and down thing and me and her doctor are trying to figure what is the issue because she doesn’t really eat that much, but I think it’s because she is over everybody’s house and the other kids are probably eating a lot of junk, I think that’s what it is because she does not eat that much at home.

Research shows that peer influence or the need to fit into peer groups often has negative effects on one’s unhealthy food consumption but depends on many factors, such as adolescents’ self-regulatory skills, influences of their parents, or the context that the influences happen [39]. In other words, in one context, peers may have a negative influence and others not, and likewise parents may have a variable influence as well. More research is needed to identify these situations and influences.

## Conclusion and implications

The Social Ecological Model highlights the complexity of fostering adolescent behavior change [40, 41]; employment of parenting strategies is just one of many influences the child’s behavior. Not only does adolescents’ individual characteristics matter but interactions with parents, siblings, and peers (interpersonal), the characteristics of home and school environment (schools and community), and societal influences, all have an interacting effect. Although most parents experienced some difficulties fostering a healthy, physically active lifestyle for their child, individual characteristics of adolescents who were overweight or obese and urban, lower social economic communities and schools provided unique challenges.

Parents in the present study struggled to be effective because myriad of negative influences; they had to deal with binge eating symptoms and the difficulties being physically active as a larger person, negative interactions of peers and their own lack of knowledge and effective parental strategies, lower quality schools and the economic difficulties faced by families living in urban and from lower social economical class. Some parents can monitor their child’s lifestyle behaviors at home, but then cannot monitor them in the broader context of the neighborhood community or schools. Some urban schools do not have quality lunch programs restricting unhealthy choices or provide physical activity opportunities for students through after school programs. The urban community often lacks physical activity infrastructures, which limit the opportunities for children and adolescents to engage in physical activity at home due to finances. In addition, the neighborhoods are not safe for children and adolescents to be physically active outside.

Parents are a key component in any healthy behavior lifestyle intervention involving adolescents who are overweight or obese. However, parents in the present study had little success in changing their children’s lifestyle behaviors. This can be attributed to five reasons: (1) A lack of coordinated effort in monitoring and promoting healthy choices between urban schools and parents at home. (2) Parents lack effective strategies for a lifestyle behavior change. Given their unique situations, many strategies for behavioral changes cannot work for their children. Further research needs to dive in deeper to explore what behavioral change strategies would work more effectively for children who are overweight or obese. Those evidence-based strategies should then be packaged and disseminated to parents of children who are overweight or obese. (3) Parents lack scientific knowledge of weight management from the perspective of energy balance. Some parents are puzzled that their children did not lose any weight by walking a couple miles per day. To lose body weight, an individual must adopt an energy balance approach. That is, energy intake must equate energy expenditure over time to maintain a stable body weight [42]. Without controlling caloric intake through eating and with moderate levels of physical activity, such as slow walking, parents cannot expect their children to lose body weight. It is suggested that parents from urban, lower social economic classes be provided with a comprehensive package of scientific information about weight management. This package shall include the concept of energy balance, information about the number of calories burned through physical activities and calories intake by eating different types of foods, and steps-by steps instruction on how to calculate the energy balance Eq. 4) Their children’s behaviors presented significant challenges for those parents in the present study. The findings showed that some children who are overweight or obese are unmotivated and lack persistence, did sneaking eating for a variety of reasons, and faced numerous peer pressure. Motivation and persistence are crucial factors for successful lifestyle behavior changes. It is well-known that a lack of social support makes it challenging for children to adhere to healthy living practices to achieve or maintain weight loss [43, 44]. Therefore, parents should be equipped with effective strategies to provide critical social support to help their children be motivated and persist in their pursuit of developing and maintaining a healthy, physically active lifestyle. 5) The need for effective parental strategies is great, especially given the challenges due to the individual constraints of adolescents who are overweight or obese (e.g., larger body size, teasing due to weight). Parents find their influence lowered as their son or daughter needs to make healthy choices in the context of negative peer influence. Compounding the issue, some adolescents who are overweight or obese exhibit binge eating symptoms, such as sneaky eating, and their motivation and persistence can waver especially given the hurdles they face, and the amount of weight needed to lose. The urban, lower social economic circumstances, however, present greater challenges to parents in the present study. Even though some parents possess the scientific knowledge or effective parental strategies to help their child, they do not have the time or money to employ them because they are working two jobs to pay the bills or in some cases, are a single parent.

Money, time, and dangerous neighborhood are unique barriers faced by parents of children who are overweight or obese from urban and lower social economic class. Without financial support, parents struggled to provide healthy food choices and even medical examination for their children. Parents lack time to play with their children. Research showed that parents ranked environmental safety as the most important factor when determining whether to let their young children play in a location [45]. Some parents did not allow their children to play in their neighborhood due to safety concerns. This limits the opportunities for children to access physical activities at home. Now more than ever, urban, lower social economical areas need safe places where adolescents have supervision and structures to be physically active. Research evidence showing that creating a supervised, safe place for children living in dangerous neighborhoods to play increased their physical activity levels [33]. This approach can provide a possible solution for obesity prevention and intervention for families from urban settings and lower social economic class. In addition, the employment of social workers or counselors to help physical activity is important to support these practitioners as they provide supervision to adolescents who are overweight or obese. Many adolescents who are overweight or obese have experienced trauma due to teasing or harassment because of their weight and size and do not have adaptive coping mechanisms. Increasing their self-efficacy and self-regulatory capabilities takes time and need to be constantly reinforced especially given the urban context.

Although children and adolescents are expected to live a healthy and physically active lifestyle, they may be unable to overcome most of the challenges and barriers identified in the present study. Those challenges and barriers include the pricy healthy food, busy family lives, parents not being able to afford taking their child to see a doctor, unsafe neighborhoods, poor physical activity infrastructure, and unhealthy environments in general related to healthy eating and physical activity. A comprehensive intervention approach targeting multiple components from multiple levels identified in the Social Ecological Model [40, 41] shall be utilized to design future intervention research on childhood obesity.

There are five limitations in the present study. First, this study was descriptive in nature. Second, we did not collect parents’ background information. Third, we used a convenience sample. Fourth, there is an absence of the adolescents’ perspectives. Including interview data from adolescents or from both adolescents and their parents could have offered additional or more nuanced insights into the challenges and barriers encountered by both adolescents and their parents. Last, there was an underrepresentation of fathers in the present study as compared to mothers. The participation was based on availability of adolescents’ parents. Few fathers participated in the interview, which was consistent with the participant rate of fathers in the literature [46]. Increasing the representation rate from fathers or including both fathers and mothers in the interviews could have offered additional or more nuanced insights into the challenges and barriers faced by both parents.

Our findings highlight the importance of understanding these barriers, which will be crucial to helping school-based and healthcare professionals, researchers, and policymakers who work with childhood overweight and obesity develop interventions and policies. Some of those barriers, such as time, money, and dangerous neighborhood, are unique challenges encountered by parents with children who are overweight or obese from urban, lower social economical class. Other barriers, such as peer pressure and developmental changes of adolescence, are common challenges faced by all parents. However, it becomes more complicated to deal with since those barriers are compounded with one another to affect the lifestyle behaviors of adolescents who are overweight or obese. These findings, in addition to those regarding the social problems children who are overweight or obese experience in schools, certainly merit attention in the school and public health literature and should be clearly placed within family-school collaboration practices for underrepresented students. A comprehensive approach must be used to develop solutions to remove those barriers. Future research shall focus on developing approaches to remove those barriers and challenges for effective obesity interventions.

## Data Availability

The datasets used and/or analysed during the current study are available from the corresponding author on reasonable request.
